# Methyl 6-azido-6-de­oxy-α-d-galactoside

**DOI:** 10.1107/S1600536811025323

**Published:** 2011-07-09

**Authors:** Janice M. H. Cheng, Shivali A. Gulab, Mattie S. M. Timmer, Bridget L. Stocker, Graeme J. Gainsford

**Affiliations:** a School of Chemical and Physical Sciences, Victoria University of Welllington, PO Box 600, Wellington, New Zealand; b Malaghan Institute of Medical Research, PO Box 7060, Wellington, New Zealand; c Carbohydrate Chemistry Group, Industrial Research Limited, PO Box 31-310, Lower Hutt, New Zealand

## Abstract

The structure of the title compound, C_7_H_13_N_3_O_5_, was solved using data from a multiple fragment crystal. The galactoside ring adopts a ^4^ *C*_1_ chair conformation. In the crystal, the molecules are linked by strong O—H⋯O hydrogen bonds, which build linkages around the screw axis of the cell in a similar way to the iodo analogue. These C-5 and C-6 packing motifs expand to *R*_2_^2^(10), *C*_2_^2^(7) and *C*_2_^2^_2_(8) motifs, as found in closely related compounds.

## Related literature

For details of the synthesis, see Cheng *et al.* (2011 ▶). For related structures, see Sikorski *et al.* (2009 ▶), Robertson & Sheldrick (1965 ▶), Zhou *et al.* (2002 ▶), Kurhade *et al.*(2011 ▶). For the iodo derivative, see: Gulab *et al.* (2010 ▶). For ring conformations, see: Cremer & Pople (1975 ▶) and for hydrogen-bond motifs, see: Bernstein *et al.* (1995 ▶). For the Hooft parameter, see: Hooft *et al.* (2008 ▶).
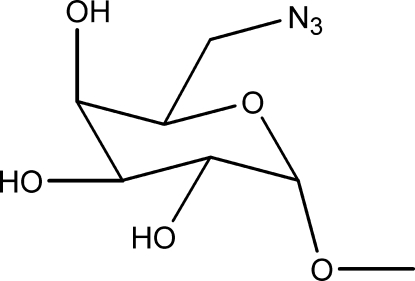


## Experimental

### 

#### Crystal data

C_7_H_13_N_3_O_5_
*M* *_r_* = 219.20
Monoclinic, 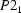

*a* = 5.8272 (5) Å
*b* = 7.8358 (6) Å
*c* = 11.0387 (10) Å
β = 102.117 (7)°
*V* = 492.81 (7) Å^3^
*Z* = 2
Cu *K*α radiation
μ = 1.09 mm^−1^
*T* = 123 K
0.20 × 0.10 × 0.02 mm

#### Data collection

Rigaku Spider diffractometer
Absorption correction: multi-scan (*ABSCOR*; Higashi, 1995 ▶) *T*_min_ = 0.581, *T*_max_ = 1.0
4122 measured reflections
1114 independent reflections
1039 reflections with *I* > 2σ(*I*)
*R*_int_ = 0.048
θ_max_ = 56.9°

#### Refinement

*R*[*F* ^2^ > 2σ(*F* ^2^)] = 0.047
*wR*(*F* ^2^) = 0.125
*S* = 1.04
1114 reflections
143 parameters
2 restraints
H atoms treated by a mixture of independent and constrained refinement
Δρ_max_ = 0.21 e Å^−3^
Δρ_min_ = −0.24 e Å^−3^
Absolute structure: Flack (1983 ▶), 491 Friedel pairs
Flack parameter: 0.1 (5)

### 

Data collection: *CrystalClear* (Rigaku, 2005 ▶); cell refinement: FSProcess in *PROCESS-AUTO* (Rigaku, 1998 ▶); data reduction: FSProcess in *PROCESS-AUTO*; program(s) used to solve structure: *SHELXS97* (Sheldrick, 2008 ▶); program(s) used to refine structure: *SHELXL97* (Sheldrick, 2008 ▶); molecular graphics: *ORTEP* in *WinGX* (Farrugia, 1997 ▶) and *Mercury* (Macrae *et al.*, 2008 ▶); software used to prepare material for publication: *SHELXL97* and *PLATON* (Spek, 2009 ▶).

## Supplementary Material

Crystal structure: contains datablock(s) global, I. DOI: 10.1107/S1600536811025323/fj2434sup1.cif

Structure factors: contains datablock(s) I. DOI: 10.1107/S1600536811025323/fj2434Isup2.hkl

Additional supplementary materials:  crystallographic information; 3D view; checkCIF report

## Figures and Tables

**Table 1:** Hydrogen-bond geometry (Å, °)

*D*—H⋯*A*	*D*—H	H⋯*A*	*D*⋯*A*	*D*—H⋯*A*
O2—H2*O*⋯O3^i^	0.84	1.91	2.742 (4)	170
O4—H4*O*⋯O2^ii^	0.81 (3)	2.00 (4)	2.774 (4)	162 (6)
O3—H3*O*⋯O4^i^	0.84	2.02	2.841 (4)	165

Symmetry codes: (i) 
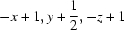
; (ii) 
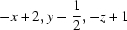
.

**Fig. 1.**
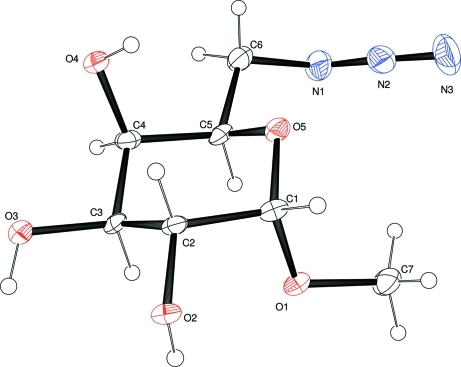
Asymmetric unit contents of (I) (Farrugia, 1997) at the 30% thermal ellipsoid level.

**Fig. 2.**
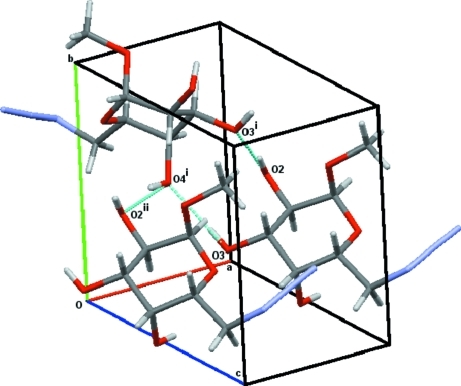
A packing view (Mercury 2.3, Macrae et al. (2008)) of the cell highlighting major hydrogen bonds (dotted). Symmetry codes: (i) 1 - x, 1/2 + y, 1 - z (ii) x - 1, y, z.

## supplementary crystallographic information

### Comment

Alkyl azidoglycosides such as the title compound (I) are versatile synthetic intermediates for the synthesis of a wide array of biologically important molecules (Cheng *et al.*, 2011; Kurhade *et al.*,2011; Zhou *et al.*,2002).

The asymmetric unit of the title compound (I) contains one independent methyl 6-deoxy-6-azido-α-D-galactoside molecule (Fig. 1). The galactoside ring (C1–C5,O5) has a ^4^C_1_ chair conformation with Q 0.581 (4) Å, θ & φ 2.5 (5) & 288 (9)° respectively (Cremer & Pople, 1975) identical to that of the iodo-analogues (Gulab *et al.*, 2010) and similar to that of the corresponding glucopyranoside 0.563 (5) Å, 4.8 (5)° & 310 (5)° (BOSLEB, Sikorski *et al.*, 2009), The absolute configurations known from the synthesis of C1(*R*), C2(*R*), C3(S), C4(*R*) and C5(S) are consistent with the weak indications given by the Hooft parameter (0.0 (2), Hooft *et al.*, (2008)), compared with the indeterminate Flack value of 0.1 (5).

Lattice binding is provided by O—H···O hydrogen bonds (Table 1), which build linkages around the b screw axis of the cell (Figure 2). This binding is notably similar to that observed for the iodo derivative (Gulab *et al.*, 2010), the bromohydrin analogue (MGALBH, Robertson & Sheldrick, 1965), and the corresponding glucopyranoside (BOSLEB). The basic motif building blocks (Bernstein *et al.*, 1995) are of C(6) & C(5) types, which combine to give ^2^*R*_2_(10), ^2^C_2_(7) & ^2^C_2_(8) motifs. Close similarity with the iodo analogue is observed: the major difference concerns the approximately doubled *c* axis length and the presence of the two additional 2_1_ screw axes in the latter orthorhombic crystal.

### Experimental

Methyl 6-azido-6-deoxy-α-*D*-galactopyranoside was prepared as described in Cheng *et al.* (2011) from methyl 6-deoxy-6-iodo-α-*D*-galactopyranoside as reported in Gulab *et al.* (2010). The title compound was recrystallized from methanol.

### Refinement

The crystals were predominantly multiple-fragment blocks up to ~0.7 *x* 0.6 *x* 0.2 mm in size. The minute crystal which was finally selected consisted of one major fragment with some minor fragments along the long (needle) axis. In addition, the data collection was partially marred by crystal movement initiated by ice buildup. The dataset arises from processing the remaining 155 screens of data after removing the affected 66 slices which were identified by the screen review statistics; this was verified by visual inspection of the screens which showed misalignment and ice rings. In the (automatic) processing, a further 209 outliers were detected and removed. High angle data was also recognized as being weak or suffering from misalignent, so the data outside the 0.92 Å shell was omitted using the SHEL command. A further 20 outlier reflections (ΔF^2^/σ(F^2^)>4.2) were removed from the refinement; some of these could be identified as being affected by ice-diffracted scattering or behind the goniometer shadow. As a result, the final dataset is significantly less than 100% complete but is adequate for defining the structure; defining the absolute configuration, known from the synthetic procedure, is not possible.

The hydroxyl atom HO4 on oxygen O4 was not placed correctly using the usual *SHELX* (AFIX 147) command, as determined by packing analysis inspection. It was placed, and then freely refined, on the basis of careful difference mapping around the O4 site and restrained to be 0.84 (3) Å from O4 using *DFIX*. The other hydroxyl H atoms were refined by automatic placement at positions indicated by a difference electron density map and their positions were constrained to refine on their parent O atoms with O–H 0.84 Å (using AFIX 147). The hydroxyl H atoms were refined with *U*_iso_(H) = 1.5*U*_eq_(O).

The methyl H atoms were constrained to an ideal geometry (C—H = 0.98 Å) with *U*_iso_(H) = 1.5*U*_eq_(C), but were allowed to rotate freely about the adjacent C—C bond. All other H atoms were placed in geometrically idealized positions and constrained to ride on their parent atoms with C—H distances of 1.00 (primary) or 0.99 (methylene) Å and and with *U*_iso_(H) = 1.2*U*_eq_(C).

### Crystal data

**Table:**

C_7_H_13_N_3_O_5_	*F*(000) = 232
*M**_r_* = 219.20	*D*_x_ = 1.477 Mg m^−^^3^
Monoclinic, *P*2_1_	Cu *K*α radiation, λ = 1.54178 Å
Hall symbol: P 2yb	Cell parameters from 4158 reflections
*a* = 5.8272 (5) Å	θ = 7.0–66.7°
*b* = 7.8358 (6) Å	µ = 1.09 mm^−^^1^
*c* = 11.0387 (10) Å	*T* = 123 K
β = 102.117 (7)°	Plate, colourless
*V* = 492.81 (7) Å^3^	0.20 × 0.10 × 0.02 mm
*Z* = 2	

### Data collection

**Table:**

Rigaku Spider diffractometer	1114 independent reflections
Radiation source: Rigaku MM007 rotating anode	1039 reflections with *I* > 2σ(*I*)
Rigaku VariMax-HF Confocal Optical System	*R*_int_ = 0.048
Detector resolution: 10 pixels mm^-1^	θ_max_ = 56.9°, θ_min_ = 7.0°
ω scans	*h* = −6→3
Absorption correction: multi-scan (*ABSCOR*; Higashi, 1995)	*k* = −8→8
*T*_min_ = 0.581, *T*_max_ = 1.0	*l* = −11→11
4122 measured reflections	

### Refinement

**Table:**

Refinement on *F*^2^	Hydrogen site location: inferred from neighbouring sites
Least-squares matrix: full	H atoms treated by a mixture of independent and constrained refinement
*R*[*F*^2^ > 2σ(*F*^2^)] = 0.047	*w* = 1/[σ^2^(*F*_o_^2^) + (0.0786*P*)^2^ + 0.1967*P*] where *P* = (*F*_o_^2^ + 2*F*_c_^2^)/3
*wR*(*F*^2^) = 0.125	(Δ/σ)_max_ < 0.001
*S* = 1.04	Δρ_max_ = 0.21 e Å^−^^3^
1114 reflections	Δρ_min_ = −0.24 e Å^−^^3^
143 parameters	Extinction correction: *SHELXL97* (Sheldrick, 2008), Fc^*^=kFc[1+0.001xFc^2^λ^3^/sin(2θ)]^-1/4^
2 restraints	Extinction coefficient: 0.020 (4)
Primary atom site location: structure-invariant direct methods	Absolute structure: Flack (1983), 491 Friedel pairs
Secondary atom site location: difference Fourier map	Flack parameter: 0.1 (5)

### Special details

**Table:**

Geometry. All e.s.d.'s (except the e.s.d. in the dihedral angle between two l.s. planes) are estimated using the full covariance matrix. The cell e.s.d.'s are taken into account individually in the estimation of e.s.d.'s in distances, angles and torsion angles; correlations between e.s.d.'s in cell parameters are only used when they are defined by crystal symmetry. An approximate (isotropic) treatment of cell e.s.d.'s is used for estimating e.s.d.'s involving l.s. planes.
Refinement. Refinement of *F*^2^ against ALL reflections. The weighted *R*-factor *wR* and goodness of fit *S* are based on *F*^2^, conventional *R*-factors *R* are based on *F*, with *F* set to zero for negative *F*^2^. The threshold expression of *F*^2^ > σ(*F*^2^) is used only for calculating *R*-factors(gt) *etc*. and is not relevant to the choice of reflections for refinement. *R*-factors based on *F*^2^ are statistically about twice as large as those based on *F*, and *R*- factors based on ALL data will be even larger.

### Fractional atomic coordinates and isotropic or equivalent isotropic displacement parameters (Å^2^)

**Table:**

	*x*	*y*	*z*	*U*_iso_*/*U*_eq_	
O1	0.9756 (5)	0.6454 (3)	0.6831 (2)	0.0291 (8)	
O2	0.8027 (5)	0.5541 (3)	0.4390 (2)	0.0276 (8)	
H2O	0.7366	0.6378	0.4647	0.041*	
O3	0.4659 (5)	0.3064 (4)	0.4887 (3)	0.0292 (8)	
H3O	0.4068	0.3842	0.4397	0.044*	
O4	0.7817 (5)	0.0839 (3)	0.6441 (3)	0.0287 (9)	
H4O	0.913 (5)	0.091 (8)	0.632 (5)	0.043*	
O5	1.1064 (5)	0.3653 (3)	0.7346 (2)	0.0286 (8)	
N1	1.1494 (9)	0.2908 (5)	0.9995 (3)	0.0449 (12)	
N2	1.3424 (9)	0.3419 (5)	0.9860 (4)	0.0449 (12)	
N3	1.5248 (10)	0.3958 (7)	0.9855 (4)	0.0634 (16)	
C1	1.0514 (9)	0.4929 (5)	0.6392 (4)	0.0263 (12)	
H1	1.1946	0.5161	0.6053	0.032*	
C2	0.8568 (7)	0.4282 (5)	0.5348 (4)	0.0241 (11)	
H2	0.9169	0.3246	0.4986	0.029*	
C3	0.6484 (8)	0.3764 (5)	0.5835 (4)	0.0248 (11)	
H3	0.5851	0.4800	0.6182	0.030*	
C4	0.7136 (8)	0.2439 (5)	0.6880 (4)	0.0251 (11)	
H4	0.5733	0.2246	0.7252	0.030*	
C5	0.9054 (8)	0.3214 (5)	0.7856 (4)	0.0320 (12)	
H5	0.8442	0.4269	0.8188	0.038*	
C6	0.9996 (9)	0.2007 (6)	0.8927 (4)	0.0342 (12)	
H6A	0.8662	0.1461	0.9203	0.041*	
H6B	1.0927	0.1096	0.8636	0.041*	
C7	1.1589 (9)	0.7300 (6)	0.7705 (4)	0.0389 (13)	
H7A	1.2933	0.7506	0.7320	0.058*	
H7B	1.2077	0.6577	0.8439	0.058*	
H7C	1.1001	0.8391	0.7951	0.058*	

### Atomic displacement parameters (Å^2^)

**Table:**

	*U*^11^	*U*^22^	*U*^33^	*U*^12^	*U*^13^	*U*^23^
O1	0.034 (2)	0.0158 (14)	0.0364 (15)	0.0004 (13)	0.0049 (15)	−0.0036 (13)
O2	0.0303 (19)	0.0160 (15)	0.0353 (16)	0.0039 (13)	0.0038 (15)	0.0044 (12)
O3	0.0261 (18)	0.0200 (14)	0.0387 (16)	−0.0024 (13)	0.0004 (13)	0.0018 (12)
O4	0.0276 (18)	0.0178 (15)	0.0398 (17)	0.0018 (15)	0.0045 (17)	−0.0030 (13)
O5	0.0264 (17)	0.0206 (14)	0.0393 (15)	−0.0028 (14)	0.0083 (15)	0.0041 (14)
N1	0.051 (3)	0.041 (2)	0.036 (2)	−0.004 (2)	−0.007 (2)	−0.0040 (19)
N2	0.046 (3)	0.039 (3)	0.042 (2)	0.003 (3)	−0.009 (3)	0.001 (2)
N3	0.039 (3)	0.080 (4)	0.061 (3)	−0.013 (3)	−0.014 (3)	0.015 (3)
C1	0.034 (3)	0.016 (2)	0.029 (2)	0.0015 (18)	0.009 (2)	0.0030 (17)
C2	0.024 (2)	0.0133 (19)	0.034 (2)	0.0015 (18)	0.005 (2)	0.0042 (17)
C3	0.026 (2)	0.0115 (19)	0.035 (2)	0.0000 (19)	0.003 (2)	−0.0067 (19)
C4	0.026 (3)	0.018 (2)	0.033 (2)	0.0044 (18)	0.009 (2)	0.0015 (19)
C5	0.039 (3)	0.014 (2)	0.039 (2)	−0.0002 (19)	−0.002 (2)	−0.0067 (19)
C6	0.040 (3)	0.024 (3)	0.036 (2)	−0.0039 (19)	0.003 (2)	0.0008 (18)
C7	0.049 (3)	0.024 (2)	0.038 (2)	−0.005 (2)	−0.005 (2)	−0.004 (2)

### Geometric parameters (Å, °)

**Table:**

O1—C1	1.395 (5)	C1—H1	1.0000
O1—C7	1.441 (5)	C2—C3	1.484 (6)
O2—C2	1.432 (5)	C2—H2	1.0000
O2—H2O	0.8400	C3—C4	1.540 (6)
O3—C3	1.435 (5)	C3—H3	1.0000
O3—H3O	0.8400	C4—C5	1.508 (5)
O4—C4	1.430 (5)	C4—H4	1.0000
O4—H4O	0.81 (3)	C5—C6	1.523 (5)
O5—C1	1.439 (5)	C5—H5	1.0000
O5—C5	1.444 (6)	C6—H6A	0.9900
N1—N2	1.232 (6)	C6—H6B	0.9900
N1—C6	1.489 (5)	C7—H7A	0.9800
N2—N3	1.144 (7)	C7—H7B	0.9800
C1—C2	1.524 (6)	C7—H7C	0.9800
			
C1—O1—C7	112.5 (3)	O4—C4—C5	112.2 (3)
C2—O2—H2O	109.5	O4—C4—C3	112.3 (3)
C3—O3—H3O	109.5	C5—C4—C3	107.0 (3)
C4—O4—H4O	110 (4)	O4—C4—H4	108.4
C1—O5—C5	112.1 (3)	C5—C4—H4	108.4
N2—N1—C6	117.2 (4)	C3—C4—H4	108.4
N3—N2—N1	173.1 (5)	O5—C5—C4	110.9 (3)
O1—C1—O5	112.3 (3)	O5—C5—C6	105.1 (4)
O1—C1—C2	108.0 (4)	C4—C5—C6	113.4 (3)
O5—C1—C2	109.8 (3)	O5—C5—H5	109.1
O1—C1—H1	108.9	C4—C5—H5	109.1
O5—C1—H1	108.9	C6—C5—H5	109.1
C2—C1—H1	108.9	N1—C6—C5	112.1 (4)
O2—C2—C3	112.7 (3)	N1—C6—H6A	109.2
O2—C2—C1	110.0 (3)	C5—C6—H6A	109.2
C3—C2—C1	110.7 (3)	N1—C6—H6B	109.2
O2—C2—H2	107.8	C5—C6—H6B	109.2
C3—C2—H2	107.8	H6A—C6—H6B	107.9
C1—C2—H2	107.8	O1—C7—H7A	109.5
O3—C3—C2	112.2 (3)	O1—C7—H7B	109.5
O3—C3—C4	108.5 (3)	H7A—C7—H7B	109.5
C2—C3—C4	111.3 (3)	O1—C7—H7C	109.5
O3—C3—H3	108.2	H7A—C7—H7C	109.5
C2—C3—H3	108.2	H7B—C7—H7C	109.5
C4—C3—H3	108.2		
			
C7—O1—C1—O5	66.9 (5)	C2—C3—C4—O4	−67.6 (4)
C7—O1—C1—C2	−172.0 (4)	O3—C3—C4—C5	179.9 (4)
C5—O5—C1—O1	60.9 (4)	C2—C3—C4—C5	56.0 (4)
C5—O5—C1—C2	−59.1 (4)	C1—O5—C5—C4	62.8 (4)
O1—C1—C2—O2	57.9 (4)	C1—O5—C5—C6	−174.3 (3)
O5—C1—C2—O2	−179.5 (3)	O4—C4—C5—O5	65.1 (4)
O1—C1—C2—C3	−67.3 (4)	C3—C4—C5—O5	−58.6 (4)
O5—C1—C2—C3	55.3 (5)	O4—C4—C5—C6	−53.0 (5)
O2—C2—C3—O3	59.4 (4)	C3—C4—C5—C6	−176.6 (4)
C1—C2—C3—O3	−177.0 (3)	N2—N1—C6—C5	−70.3 (5)
O2—C2—C3—C4	−178.8 (3)	O5—C5—C6—N1	71.0 (4)
C1—C2—C3—C4	−55.2 (4)	C4—C5—C6—N1	−167.6 (4)
O3—C3—C4—O4	56.3 (5)		

### Hydrogen-bond geometry (Å, °)

**Table:**

*D*—H···*A*	*D*—H	H···*A*	*D*···*A*	*D*—H···*A*
O2—H2O···O3^i^	0.84	1.91	2.742 (4)	170
O4—H4O···O2^ii^	0.81 (3)	2.00 (4)	2.774 (4)	162 (6)
O3—H3O···O4^i^	0.84	2.02	2.841 (4)	165

Symmetry codes: (i) −*x*+1, *y*+1/2, −*z*+1; (ii) −*x*+2, *y*−1/2, −*z*+1.
